# Editorial: Global excellence in ethnopharmacology: North and South America

**DOI:** 10.3389/fphar.2024.1488930

**Published:** 2024-09-17

**Authors:** Sol Cristians, Rachel Mata

**Affiliations:** ^1^ Botanical Garden, Institute of Biology, National Autonomous University of Mexico, Mexico City, Mexico; ^2^ Faculty of Chemistry, National Autonomous University of Mexico, Mexico City, Mexico

**Keywords:** ethnopharmacology, South America, North America, medicinal plant, medicinal fungi, quality control

The Research Topic entitled *Global Excellence in Ethnopharmacology: North and South America* summarizes the contributions of specialized scientists and health officers from North, Central, and South America. For many reasons, most commonly due to continuous migrations from south to north, many indigenous plants have crossed the frontiers of American countries and are now Pan-American. All the works presented had roots in medicinal agents traditionally employed in the region. The Research Topic shows the multiple approaches to ethnopharmacological research, reflecting a biodiverse continent with much to offer. The articles comprise bioprospecting, the relevance of national and Pan-American plant-derived natural product libraries, and the need for homogeneous quality consistency of Pan-American medicinal plants.

Our Research Topic comprises five papers:

*Honeybee pollen from Southern Chile: phenolic profile, antioxidant capacity, bioaccessibility, and inhibition of DNA damage* Bridi et al. is a pioneering effort by Chilean researchers. They have, for the first time, characterized honeybee pollen according to its botanical origin, phenolic composition, and antioxidant activity. This research shows the potential for developing future treatments for preventing gastric and/or intestinal cancer. Notably, the introduced species *Brassica rapa* L. (Brassicaceae), *Lotus pedunculatus* Cav., and *Ulex europaeus* L. (Fabaceae) were found to predominate in all honeybee pollen analyzed, while the native species *Buddleja globosa* Hope (Scrophulariaceae) and *Luma apiculata* (DC.) Burret (Myrtaceae), *Embothrium coccineum* J.R. Forst. & G. Forst. (Proteaceae), and *Eucryphia cordifolia* Cav. (Cunoniaceae) appeared less frequently.

*Exploring the chemical and pharmacological variability of Lepidium meyenii: a comprehensive review of the effects of Maca* Carpio et al. carried out by experts from Peru, Costa Rica, and the United States, brings together phytochemical information and preclinical and clinical evidence of the uses and pharmacological effects of *L. meyenii*. This resource is marketed worldwide and represents one of the best examples of the globalization of traditional medicine. Maca is indigenous to Peru and Bolivia, where it has been used for more than 2000 years as medicinal and crop food. Nowadays, Maca is commercialized across the continent and in Europe. According to verified Market reports, Maca Products Market size was valued at USD 55.54 billion in 2023 and is projected to reach USD 77.09 billion by 2030 (https://www.verifiedmarketreports.com/download-sample/?rid=442646).

*On the importance for drug discovery of a transnational Latin American database of natural compound structures.* Thomson The importance of a transnational Latin American database of natural compound structures for drug discovery is highlighted in this paper. It summarizes current advances in chemical space expansion aimed at natural products from Latin America, with a focus on target structural prediction and chemical library constructions. This research is expected to energize drug discovery in the region and promote the development of new synthetic chemical and biosynthetic strategies.

*Discovery of inhibitors of protein tyrosine phosphatase 1B contained in a natural products library from Mexican medicinal plants using a combination of enzymatic and in silico methods* Díaz-Rojas et al. is a novel study carried out by Mexican researchers who analyzed a total of 423 Mexican natural products from the BIOFACQUIM database. They performed a multi-stage screening that allowed the proposal of three compounds intended for drug optimization analysis to develop new leads for diabetes drug development, targeting protein tyrosine phosphatase 1B, an essential enzyme in the pathophysiology of diabetes and the homeostasis of glucose. This article provides a contemporary tool for bioprospecting and exemplifies the proposal of the work by T. Thomson.

*A U.S. Pharmacopeia (USP) Overview of Pan American Botanicals Used in Dietary Supplements and Herbal Medicines* Upton et al. provides an overview of the state of botanical dietary supplements and herbal medicines in American countries with a focus on the regulatory status of herbal products, the development of national quality and research initiatives, and policies related to agriculture conservation and sustainability, among other topics. Like Maca, many other American indigenous plants are employed throughout the continent, and the unavoidable globalization of As Maca, many other American indigenous plants are employed throughout the continent. Thus, the unavoidable globalization of these plants brings forth the need to implement health policies, including regularization, quality control, and efficacy studies. In response, United States Pharmacopeia is developing a robust body of monographs to guide industry and regulators in ensuring the quality and safety of botanical ingredients used in dietary supplements and herbal medicines in the Pan-American region.

